# From Genotype to Phenotype: Linking Bioinformatics and Medical Informatics Ontologies

**DOI:** 10.1002/cfg.181

**Published:** 2002-10

**Authors:** Ele Holloway

**Affiliations:** 1 European Bioinformatics Institute Wellcome Trust Genome Campus Hinxton Cambridge CB10 1SD UK

## Abstract

A small group of around 40 people came together at the Chancellors Conference Centre in Manchester for the Ontologies Workshop, chaired by Alan Rector and Robert Stevens. The workshop was, rather strangely, spread over 2 half days. In hindsight, this programme worked very well as it gave people the opportunity to chat over a drink on the Saturday evening and share ideas, before launching into the second half on the following day. The participants were from various walks of life, all with a common interest in finding out more about ontologies and promoting collaborations between the medical informatics and bioinformatics ontology communities.


**Comparative and Functional Genomics**
*Comp Funct Genom* 2002; **3**: 447–450.
Published online 29 August 2002 in Wiley InterScience (www.interscience.wiley.com). **DOI:** 10.1002/cfg.181

**Feature**

### Meeting Review: From Genotype to Phenotype: Linking Bioinformatics and Medical Informatics Ontologies

Manchester, UK, 23–24 March 2002

Ele Holloway*
*European Bioinformatics Institute, Wellcome Trust Genome Campus, Hinxton, Cambridge CB10 1SD, UK*

*Correspondence to: Ele Holloway, European Bioinformatics Institute, Wellcome Trust Genome Campus, Hinxton, Cambridge CB10 1SD, UK.. E-mail: ele@ebi.ac.uk*

Received: 27 May 2002
Accepted: 6 June 2002

### Abstract

**A small group of around 40 people came together at the Chancellors Conference Centre in Manchester for the Ontologies Workshop, chaired by Alan Rector and Robert Stevens. The workshop was, rather strangely, spread over 2 half days. In hindsight, this programme worked very well as it gave people the opportunity to chat over a drink on the Saturday evening and share ideas, before launching into the second half on the following day. The participants were from various walks of life, all with a common interest in finding out more about ontologies and promoting collaborations between the medical informatics and bioinformatics ontology communities. Copyright © 2002 John Wiley & Sons, Ltd.**

**Keywords:** **ontology; controlled vocabulary; genotype; phenotype; bioinformatics; medical informatics**

### Introduction

There are different ideas of what really makes an ontology, but the common factor is that they are all created as a container to capture knowledge or information about a concept, organism, process, etc. — the list is endless. Ontologies are a hierarchical structuring of knowledge subcategorized by their relevant qualities; controlled vocabularies in the form of words or phrases are used in ontologies to bring different meanings or synonyms together under one clear term. By using controlled vocabularies, we avoid using free text and make our information understandable by computers. This makes the task of parsing the information more attainable, and can allow us to gain a shared understanding of disparate datasets through the use of ontologies.

This conference brought together not only those interested in the biology behind the ontologies, but also the bioinformaticians wanting to develop the tools to take things further and at a greater speed.

**Robert Stevens (University of Manchester)** started the proceedings by stating that in this post-genomic phase we are no longer interested in the smaller picture of single proteins; we are now looking at populations of molecules in relationship to their genotype and phenotype. He asked colleagues of his, in advance of the workshop, what they understood by the terms 'genotype' and 'phenotype'. He found that there was a reasonably tight understanding of 'genotype' as relating to regions of nucleic acid and involving alleles, whereas the term 'phenotype' led to a vast array of examples, such as eye colour, Mendel's pea experiments, and disease. In general, bioinformatics ontologies have described the genotype of organisms, and phenotype has been covered more by the medical informatics ontologies (Robert Stevens Bio-ontology page: **http://img.cs.man.ac.uk/stevens/ontology.html**). Robert hopes that the linking of these ontologies

Copyright © 2002 John Wiley & Sons, Ltd.

can bring the two fields together in some interesting collaborations.

Many questions were likely to arise from this meeting, but as food for thought he put forward the questions: What are the roles of ontologies? What can be covered in the span of genotype to phenotype? Can we re-use ontologies, e.g. use an existing anatomy ontology as a template for a new anatomy ontology for another species?

The use of controlled vocabularies to standardize and provide consistency in databases was a common theme in the talks. As more genomes are studied, and sequenced faster than before, there is a greater need for the glut of data being produced to be managed efficiently, allowing researchers to query the data more effectively. Agreeing on terms to use is a vast task but will ultimately lead to users knowing the best way to query their data and other people's. The use of different synonyms, e.g. in the case of gene names, can cause data to be 'lost' in databases, so using a standard gene name makes sense. This is what the HUGO Gene Nomenclature Committee (**http://www.gene.ucl.ac.uk/nomenclature**) is working on. **Ruth Lovering (HUGO Gene Nomenclature Committee, UCL)** described how the HGNC make sure that unique and meaningful gene symbols are assigned, promoting standardization and consistency when describing genes. When she presented their work, the updated version of their guidelines was in press, but the article has since been published (Wain *et al.* 2002). Once they have assigned a gene name and gene symbol, the information is stored in the Genew database (**http://www.gene.ucl.ac.uk/cgi-bin/nomenclature/searchgenes.pl**), which also receives automatic updates from LocusLink, SWISS-PROT, GDB and MGD. Genew contains over 14 000 active gene symbols, is user-friendly and easy to query.

The importance of consistent gene naming, or at least keeping on top of gene name synonyms, was clearly illustrated in **Joyce Mitchell's (National Library of Medicine, USA)** presentation. She has been examining the coverage of the Unified Medical Language System (UMLS; UMLS and Gene Ontology navigators: **http://etbsun2.nlm.nih.gov:8000/**), GO and LocusLink-OMIM and the problems faced finding terms stored in different resources when there are so many synonyms being used. She presented a multitude of slides showing the statistical results of her searches, which essentially showed that a significant number of gene entries were not being retrieved. The main reason behind the failure to find many genes in these databases was because no single source contained all gene names and synonyms. Sometimes the gene names have comments attached to the official name, which prevents their retrieval. The message coming through from her work was that improvements in gene naming, and the storage of more information on each gene, are required to solve these problems.

**Anne Westcott (AstraZeneca Pharmaceuticals)** presented the application of genotyping in gene discovery and drug response typing. At present, the whole process of target (gene) identification, candidate drug development, concept testing, marketing, etc. can take 7–8 years; understandably they want to reduce this. The recent discovery and mapping of a massive number of single nucleotide polymorphisms (SNPs) and microsatellites (that serve as genetic markers), and the ability to collect and archive DNA from clinical trial subjects is bringing the concept of medicines tailored to the individual within reach. Using these resources could help pharmaceutical companies to develop compounds that will be effective in the majority of the population, or to identify the subset of the population for whom a particular drug will be effective and those in which it will cause side-effects. For this to happen, they need larger collections of DNA, better-characterized phenotypes, pedigree data where appropriate and, rather importantly, reproducible results. This approach is going to generate a large amount of trait-based information that needs to be stored alongside sequence data to allow comparisons between individuals or groups.

**Dieter Maier (Biomax Informatics AG, Germany: http://www.biomax.de/)** presented the FunCat controlled vocabulary, which covers biological processes and molecular functions of proteins in prokaryotes, unicellular eukaryotes, plants and animals. There are separate phenotype catalogues to contain the information for normal and mutated forms, as they do not want to mix these. FunCat is hierarchically organized and has been used in the manual and automated annotation of a range of species.

The work of the Gene Ontology (GO) group was mentioned by several of the speakers. This

Copyright © 2002 John Wiley & Sons, Ltd.

was often because their groups are part of the GO Consortium (**http://www.geneontology.org**) but mainly to acknowledge the great work being carried out. **Midori Harris (European Bioinformatics Institute)** explained that the GO project covers three ontologies: molecular function, biological process and cellular component. In building these ontologies to describe gene products and the various aspects of molecular biology, they are using controlled vocabularies that will, in turn, help in developing tools to query the ontologies, and add to them, as more is known.

**Judith Blake (Jackson Laboratory, USA)** from the Mouse Genome Informatics group (Mouse Genome Informatics: **http://www.informatics.jax.org**), who are part of the GO Consortium (**http://www.geneontology.org**), outlined three projects being undertaken: Mouse Genome Database Project (MGD), Gene Expression Database Project (GXD), and Mouse Genome Sequence Project (MGS). She highlighted their system as being a comprehensive resource containing details at the molecular level, plus information pertaining to normal and dysfunctional phenotypes, for the mouse. The relationships between these phenotypes and those observed in humans are also stored. Their phenotype ontology building is occurring whilst annotation of the mouse data is still ongoing and they support the use of controlled vocabularies and furthering the scope of ontologies. They are also in collaboration with the MRC and Edinburgh Mouse Atlas Project (EMAP), who are aiming to provide nomenclature for the parts of the mouse anatomy from fertilization to adult stage (Standard Anatomical Nomenclature Database: **http://genex.hgu.mrc.ac.uk/Databases/Anatomy/**), and provide 3-D anatomy reconstructions to view. **Albert Burger (Heriot-Watt University)** is a member of the team working on EMAP and the Edinburgh Mouse Atlas Gene Expression Database Project (EMAGE: **http://genex.hgu.mrc.ac.uk/**). He showed slides demonstrating the anatomy browser and then went on to explain the thinking behind the building of the anatomy ontology and the Edinburgh Anatomy Mapping System (EdAMS). EdAMS is a cross-species embryo anatomy system that stores confirmed mappings between tissues of different species and will suggest potential mappings, based on various rules, which need to be checked by editors to decide whether to reject or accept them.

An ontology for craniofacial abnormality research was presented by **Peter Hammond (University College London)**. He showed some fantastic, lifelike 3-D images that are stored as a record of a patient's morphology. These images are needed to fully capture a patient's features from all views. There is a need to re-scan over time due to natural phenotypic changes, such as in the case of a child ageing, or after surgery, which often causes a dramatic craniofacial change. In addition to an image ontology they need ontologies to capture information on the syndrome, the genetics of the patient, plus any other relevant data.

**Fouzia Moussouni (INSERM U522 and Medical Informatics Laboratory, France)** is working on liver disorders relating to the metabolism of iron. She is using microarrays to compare normal and pathologic samples. She stressed the need for an integrated gene expression warehouse to bring together medical, genome and array data. She made reference to the current standards in place which can pave the way forward: minimum information about a microarray experiment (MIAME: Brazma *et al.*, 2001); GO, an ontology for molecular biology and genomics; and UMLS, an aid for health professionals and researchers to use biomedical information from different sources.

For small research groups the storage and mining of microarray data is becoming a problem. One possible solution is to use a public database. **Philippe Rocca-Serra (European Bioinformatics Institute)** presented the ArrayExpress database (**http://www.ebi.ac.uk/microarray/ArrayExpress/**), which is just such a repository, designed for gene expression data. Submission of data can either be via MIAMExpress (**http://www.ebi.ac.uk/microarray/MIAMExpress/**) or a direct submission in MAGE-ML format, an XML file format that adheres to a specified content and order for microarray gene expression experiments. The data held in ArrayExpress is of a particular format and is curated to ensure consistency in the terms used (controlled vocabulary) across microarray experiments to allow easy access to information. MIAME (Brazma *et al.*, 2001), developed by the Microarray Gene Expression Data (MGED) group (**http://www.mged.org**), is a specification of the minimum information required from a microarray experiment to enable a researcher to be able to duplicate the experiment in his/her laboratory. The MIAME requirements are, in brief: experimental design, array design,

Copyright © 2002 John Wiley & Sons, Ltd.

samples, hybridizations, measurements and normalization controls. The information is annotated with the use of ontologies, where possible, and at present the MGED ontology working group is developing specific ontologies for describing experimental conditions in gene expression experiments. Where ontologies are not available, new ones will have to be developed.

**Stuart Aitken (University of Edinburgh)** presented the Rapid Knowledge Formation (RKF) project (**http://www.aiai.ed.ac.uk/~stuart/RKF/**), which aims to develop tools to enable expert biologists to construct an ontology by entering their knowledge into a knowledge-based system. Biologists took part in a pilot project to test the tools available; the knowledge they had to formalize was from a chapter in *Essential Cell Biology*, by Alberts *et al.* (1998). The results were evaluated and the ontology was revised.

**Alan Rector (University of Manchester)** from the Medical Informatics Group (**http://www.cs.man.ac.uk/mig**), talked about logic-based ontologies, OIL and DAML+OIL being modern examples. Knowledge can be broken down into smaller parts that can, with the correct terms linking them, be understandable by both humans and computers. There is still a long way to go and not everything can be expressed in this way, but logic-based ontologies can be used to a certain extent to model biology.

### Conclusion

It was clear from the presentations and from talking to people at the meeting that there is a common goal to use ontologies and to develop new ones where a void exists. Delegates were also keen to use controlled vocabularies, as they know that consistency in annotation is needed to make data mining more efficient and to gain a common understanding.

### References

Alberts B, Bray D, Johnson A, *et al.* 1998. *Essential Cell Biology*. Garland Science: New York.
ArrayExpress: **http://www.ebi.ac.uk/microarray/ArrayExpress/**
Biomax Informatics AG: **http://www.biomax.de/**
Brazma A, Hingamp P, Quackenbush J, *et al.* 2001. Minimum information about a microarray experiment (MIAME) — toward standards for microarray data. *Nature Genet* **29**(4): 365–371.
Genew: **http://www.gene.ucl.ac.uk/cgi-bin/nomenclature/searchgenes.pl**
GO Consortium: **http://www.geneontology.org**
HUGO Gene Nomenclature Committee: **http://www.gene.ucl.ac.uk/nomenclature**
Medical Informatics Group: **http://www.cs.man.ac.uk/mig**
MGED Group: **http://www.mged.org**
MIAMExpress: **http://www.ebi.ac.uk/microarray/MIAMExpress/**
Mouse Atlas and Gene Expression Database Project: **http://genex.hgu.mrc.ac.uk/**
Mouse Genome Informatics: **http://www.informatics.jax.org**
Rapid Knowledge Formation: **http://www.aiai.ed.ac.uk/~stuart/RKF/**
Robert Stevens Bio-ontology page: **http://img.cs.man.ac.uk/stevens/ontology.html**
Standard Anatomical Nomenclature Database: **http://genex.hgu.mrc.ac.uk/Databases/Anatomy/**
UMLS and Gene Ontology navigators: **http://etbsun2.nlm.nih.gov:8000/**
Wain HM, Bruford EA, Lovering RC, *et al.* 2002. Guidelines for human gene nomenclature. *Genomics* **79**(4): 464–470.

Copyright © 2002 John Wiley & Sons, Ltd.
